# HDAC4 Regulates the Proliferation, Differentiation and Apoptosis of Chicken Skeletal Muscle Satellite Cells

**DOI:** 10.3390/ani10010084

**Published:** 2020-01-04

**Authors:** Jing Zhao, Xiaoxu Shen, Xinao Cao, Haorong He, Shunshun Han, Yuqi Chen, Can Cui, Yuanhang Wei, Yan Wang, Diyan Li, Qing Zhu, Huadong Yin

**Affiliations:** Farm Animal Genetic Resources Exploration and Innovation Key Laboratory of Sichuan Province, Sichuan Agricultural University, Chengdu 611130, Sichuan, China; zhaojing@stu.sicau.edu.cn (J.Z.); shenxiaoxu@stu.sicau.edu.cn (X.S.); caoxinao@stu.sicau.edu.cn (X.C.); hehaorong@stu.sicau.edu.cn (H.H.); hanshunshun@stu.sicau.edu.cn (S.H.); chenyuqi@stu.sicau.edu.cn (Y.C.); cuican123@stu.sicau.edu.cn (C.C.); weiyuanhang@stu.sicau.edu.cn (Y.W.); as519723614@163.com (Y.W.); diyanli@sicau.edu.cn (D.L.); zhuqing@sicau.edu.cn (Q.Z.)

**Keywords:** HDAC4, SMSCs, proliferation, differentiation, apoptosis

## Abstract

**Simple Summary:**

Histone Deacetylase 4 (HDAC4) plays a critical role in cell proliferation and differentiation, but the function of HDAC4 in the skeletal muscle satellite cells (SMSCs) of chickens is still unknown. Here, we demonstrated that knockdown of HDAC4 inhibits the proliferation and differentiation of chicken SMSCs but has no significant effect on its apoptosis. These results suggest that HDAC4 has an essential role in skeletal muscle growth and in the development of chicken.

**Abstract:**

The development of skeletal muscle satellite cells (SMSCs) is a complex process that could be regulated by many genes. Previous studies have shown that Histone Deacetylase 4 (HDAC4) plays a critical role in cell proliferation, differentiation, and apoptosis in mouse. However, the function of HDAC4 in chicken muscle development is still unknown. Given that chicken is a very important meat-producing animal that is also an ideal model to study skeletal muscle development, we explored the functions of HDAC4 in chicken SMSCs after the interference of HDAC4. The results showed that HDAC4 was enriched in embryonic skeletal muscle, and it was highly expressed in embryonic muscle than in postnatal muscles. Meanwhile, knockdown of HDAC4 could significantly inhibit the proliferation and differentiation of chicken SMSCs but had no effect on the apoptosis of SMSCs as observed in a series of experiment conducted in vitro. These results indicated that HDAC4 might play a positive role in chicken skeletal muscle growth and development.

## 1. Introduction

Skeletal muscle is an important component of animal body that plays a vital role in initiating movement, maintaining homeostasis, and supporting respiration [1]. Meanwhile, the skeletal muscle is an important economic trait in meat-producing animals. For birds, the number of muscle fibers is determined at birth and postnatal muscle growth is due to hypertrophy or an increase in muscle fiber size, which is accompanied by the proliferative activity of the skeletal muscle satellite cell (SMSC). SMSCs are the kind of pluripotent stem cells that with the ability to proliferate and differentiate could be transformed from a quiescent state into mature muscle fibers under appropriate stimulation [2,3]. Thus, the repair and regeneration of skeletal muscles after injury mainly depends on SMSCs [2,4]. It is well-known that growth of SMSCs is regulated by a complex set of biological processes, including many genes [5,6].

Histone deacetylase (HDACs) are significant enzymes that play an important role in the modification of chromosome structure and controlling of gene expression via regulation of the acetylation status of histones and non-histones [7,8]. The HDAC family are composed of 18 enzymes that are classified into four groups (I to IV), according to function and the specifications of their catalytic mechanisms [9,10,11]. Interestingly, among of them, class IIa HDACs are unique because they have a special N-terminal domain and can interact with various transcription factors, such that class IIa HDACs can effectively regulate transcription [9,12].

Histone deacetylase 4 (HDAC4) is a key member of class IIa HDACs that does not directly bind to DNA, but interacts with MEF2 transcription factors [13]. HDAC4 is widely expressed in multiple tissues, especially in the brain, heart, and skeletal muscles [11,13,14]. In mice, HDAC4 alleviated skeletal muscle atrophy caused by denervation [15,16]. Cohen et al. have demonstrated that HDAC4 is a key link between nerve activity and muscle gene expression, enabling nerve stimulation in muscles and causing muscle remodeling in C57/BL mice [17]. Additionally, HDAC4 phosphorylation and subcellular reorientation are involved in the maintenance and recombination of muscle fiber function in C2C12 myoblasts [18]. Meanwhile, HDAC4 also promoted C2C12 myoblasts proliferation and inhibited myogenic differentiation [19,20].

Although there are ample evidences that HDAC4 plays an important role in skeletal muscle development, it has never been reported in chicken muscle. In this study, we investigated the role of HDAC4 in the proliferation, differentiation and apoptosis of chicken SMSCs by knocking down the expression of HDAC4.

## 2. Material and Methods

### 2.1. Tissue Sample Preparation

The animal experiment was conducted with the permission of the Committee on Experimental Animal Management of Sichuan Agricultural University, permit number 2019-12.

In this study, heart, liver, spleen, lung, kidney, breast muscle, leg muscle, brain, intestine and abdominal fat of three ROSS-308 broiler chickens were collected and snap-frozen in liquid nitrogen and stored at −80 °C freezer until use. In addition, we collected breast muscle of Ross-308 broilers from the embryonic stage of 10 days (E10) to 35 days post-hatching (P35). Three chickens were used at each time-point. All chickens were obtained from the Sichuan Yuguan Agriculture Co., Ltd. (Suining, Sichuan, China).

### 2.2. Isolation, Culture, and Transfection of Chicken SMSCs

The breast muscles of 5-day-old Ross-308 broilers were used to isolate SMSCs, as described by a previous study [21]. SMSCs were cultured in Dulbecco’s Modified Eagle Medium (DMEM; Sigma, St. Louis, MO, USA), supplemented with 10% fetal bovine serum (FBS; Gibco, Grand Island, NY, USA) and 1% penicillin–streptomycin (Solarbio, Beijing, China), and cultured in a humid environment of 37 °C and 5% CO_2_. Transfections were carried out when the cells approximately reached 80%~90% confluence, using a 1:1 ratio of DNA (μg)/Lipofectamine TM3000 (μL) (Invitrogen, Carlsbad, CA, USA). Three small interfering RNAs (si-HDAC4-1107, si-HDAC4-1709, and si-HDAC4-2536) for HDAC4 and negative siRNA (Table 1) were designed and synthesized (GenePharma, Shanghai, China) to transfect the SMSCs.

### 2.3. RNA Exaction, cDNA Synthesis, and Quantitative Real-Time PCR (qPCR)

Total RNAs were extracted from cells by a TRIzol reagent (Invitrogen), and the quality and concentration were evaluated for optical density 260/280 ratio using a Nanodrop 2000 spectrophotometer, and reverse transcriptions were performed by the PrimeScript™ RT Reagent Kit with the gDNA Eraser Kit (TaKaRa Biotechnology, Tokyo, Japan), following the manufacturer’s instructions. qPCR primers (Table 2) were designed by Primer Premier 5 software. The reaction system was 10 μL, including 5 μL of SYBR Green Premix Ex Taq (TaKaRa), 3μL of RNasefree H_2_O (Tiangen, Beijing, China), 0.5 μL of forward primer, 0.5 μL of reverse primer, and 1 μL of cDNA. The reaction conditions were as follows—95 °C for 3 min, followed by 95 °C for 10 s, the primer-specific annealing temperature for 20 s, and 72 °C for 20 s; these were then repeated 40 times. The β-actin gene was used as an internal control. Each sample was assayed in triplicates.

### 2.4. Cell Proliferation Assay 

Cell counting kit-8 (CCK-8) and 5-Ethynyl-20-Deoxyuridine (EdU) incorporation assays were used to evaluate the cell proliferation activity. For the CCK-8 assay, the cells were seeded in 96-well plates, and after 12, 24, 36, and 48 h of transfection, 10 μL of cck-8 (Bestbio, Shanghai, China) was added and incubated for 1 h at 37 °C, and the absorbance at 450 nm was measured using a Microplate Reader (Thermo, former Savant, MA, USA). Each treatment group had eight independent replicates. For the EdU assay, SCs were seeded in 96-well plates and transfected 36 h later, 100 μL of 50 μM EdU from Cell-LightTM EdU Apollo 567 In Vitro Kit (Ribobio, Guangzhou, China) was added to each well and incubated at 37 °C for 3 h. After that, the cells were fixed and stained with Apollo and Hoechst 33,342, according to the manufacturer’s instructions, and each treatment group had six independent replicates. The number of stained cells was counted by the Image-Pro Plus 6.0 software (Media Cybernetics, Bethesda, MD, USA).

### 2.5. Analysis of the Cell Cycle

SMSCs were plated in 6-well plates, after 48 h of transfection, the cells were collected and fixed with 70% ethanol, overnight at 4 °C. The cells were then incubated with 500 μL PI/RNase Staining Buffer Solution (BD Biosciences, San Jose, CA, USA). Cell cycle analysis was conducted by flow cytometry.

### 2.6. Analysis of Cell Apoptosis

SMSCs were plated in 6-well plates and collected 48 h after transfection. Then, the cells were stained using an Annexin V-FITC apoptosis assay kit (Beyotime, Shanghai, China). Finally, the cell apoptosis rate was evaluated by flow cytometry.

### 2.7. Immunofluorescence

SMSCs were seeded in 12-well plates. After 72 h of transfection, the cells were fixed with 4% paraformaldehyde for 30 min, then washed with PBS for three times, each time for 5 min. Then, it was permeated with 0.1% Triton X-100 for 20 min and blocked with goat serum for 30 min. The diluted primary antibody MYHC (Santa Cruz, Heidelberg, Germany) was added and incubated overnight in 4 °C refrigerator. The next day, the cells were washed three times with PBSfor 5 min each time, and incubated with fluorescence secondary antibody at 37 °C for 1 h. The nucleus was stained with 40, 6-diamidino-2-phenylindole (DAPI, Beyotime, Shanghai, China) for 5 min. The required images were observed and collected by a fluorescence microscope. The area of stained cells was calculated by Image-Pro Plus 6.0 software.

### 2.8. Western Blotting

SMSCs were seeded in 6-well plates and transfected with different treatment groups. Total proteins were extracted using lysis buffer and the concentration was measured by a bicinchoninic acid (BCA) protein assay kit (BestBio, Shanghai, China). Subsequently, proteins were separated by SDS-PAGE and transferred to polyvinylidene fluoride (PDVF) membranes (Millipore Corporation, Billerica, MA, USA). The membranes were blocked with Quickblock closed solution (Beyotime, Shanghai, China) for 1 h at room temperature and incubated overnight at 4 °C, with primary antibodies specific for anti-MYHC (Santa Cruz Biotechnology, Dallas, TX, USA, 1:1000), anti-caspase3 (Abcam, Cambridge, UK, 1:1000), anti-HDAC4 (Zenbio, Chengdu, China, 1:1000) or anti-β-tubulin (Zenbio, Chengdu, China, 1:1000). Then, the membranes were washed three times with washing buffer (Beyotime, Shanghai, China). Next, the PDVF membranes were incubated for 1 h at room temperature with horseradish peroxidase-conjugated secondary antibodies (Santa Cruz Biotechnology, 1:1000). Finally, protein bands were detected using the chemiluminescence (ECL) system (Beyotime, Shanghai, China) and analysis was done using the ImageJ software (National Health Institute, Bethesda, MD, USA). β-tubulin serves as the internal control of the experiment and each treatment group had three independent replicates.

### 2.9. Statistical Analysis

All results are expressed by means ± standard error (SEM). For two group comparison analysis, statistical significance of differences between means was analyzed by unpaired Student’s *t*-test. For multiple comparison analysis, data were analyzed by one-way ANOVA analysis using SPSS 20.0 (SPSS Inc., Chicago, IL, USA), the significant levels were set at * *p* < 0.05, ** *p* < 0.01, and ^a,b^ *p* < 0.05.

## 3. Results

### 3.1. Expression Pattern of HDAC4 in Chickens

**Figure 1:**
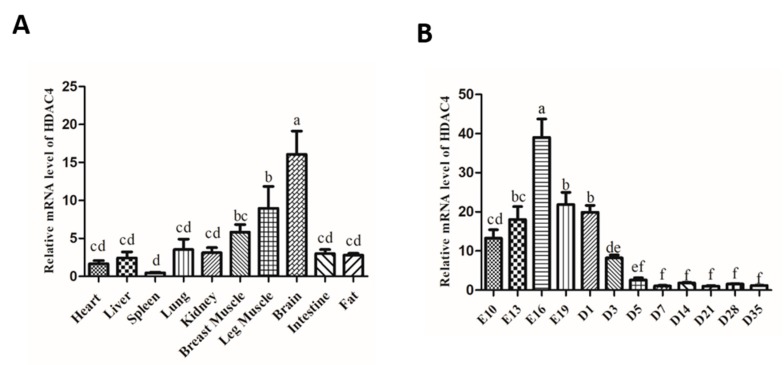
Expression pattern of HDAC4 gene in chickens. (**A**) The HDAC4 mRNA expression level was measured by qPCR in different tissues and organs of chicken. (**B**) The expression level of HDAC4 in breast muscle during E10 to D35 was detected by qPCR. In all panels, the values represent mean ± SEM from three independent experiments. ^a,b^ *p* < 0.05.

To explore the mRNA expression pattern of HDAC4 in different tissues of chicken, the mRNA level of HDAC4 in heart, liver, spleen, lung, kidney, breast muscle, leg muscle, brain, intestine, and abdominal fat were detected by qPCR. The results showed that HDAC4 was primarily expressed in the brain, and followed in breast muscle and leg muscle (Figure 1A). In addition, we measured the expression of HDAC4 in breast muscle at different developmental time-points and found that the expression of HDAC4 gradually decreased from the embryonic stage to after birth (Figure 1B). These results suggest that HDAC4 might play an important regulatory role in skeletal muscle formation.

### 3.2. Confirmation of Interference Efficiency of HDAC4

**Figure 2:**
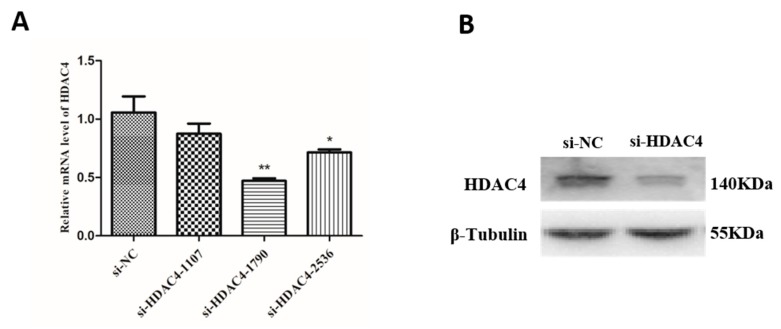
Confirmation of interference efficiency of the HDAC4 gene. (**A**) The relative mRNA level of HDAC4 in skeletal muscle satellite cells (SMSCs) after being transfected with three siRNA (si-HDAC4-1107, si-HDAC4-1709, and si-HDAC4-2536) and si-NC. (**B**) The protein expression level of HDAC4 after interference by si-HDAC4 was detected by western blotting. In all panels, the values represent mean ± SEM from three independent experiments. * *p* < 0.05; ** *p* < 0.01.

To investigate the effect of HDAC4 on the growth and development of SMSCs, three small interfering RNAs (si-HDAC4-1107,si-HDAC4-1709 and si-HDAC4-2536) were transfected into SMSCs, and si-HDAC4-1709 had the highest interference efficiency than the other two groups (Figure 2A). Next, the protein expression level of HDAC4 was evaluated by western blotting. We found that compared to the si-NC group, the protein level of HDAC4 was significantly decreased after transfected si-HDAC4-1709 (Figure 2B). Thus, si-HDAC4-1709 was selected for the subsequent research and was named si-HDAC4.

### 3.3. Knockdown of HDAC4 Inhibits the Proliferation of Chicken SMSCs

**Figure 3:**
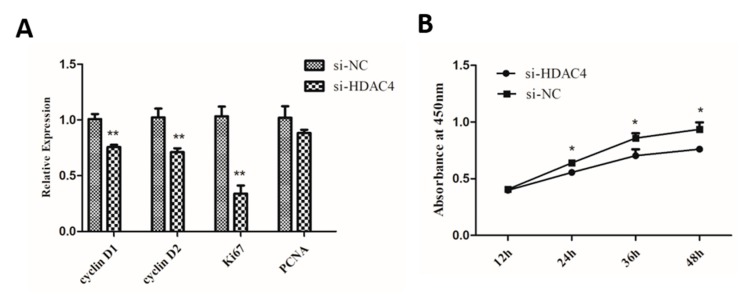

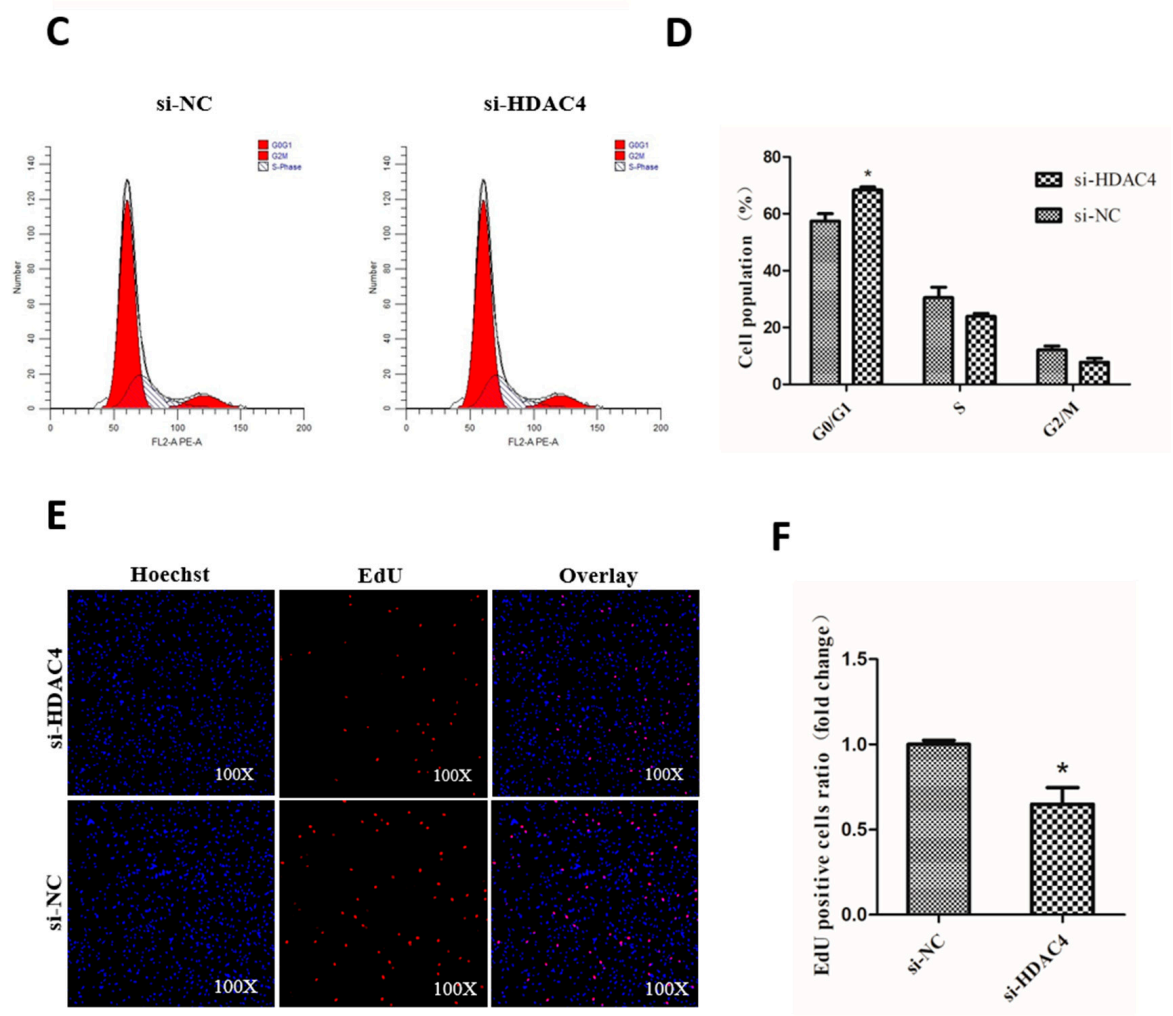
Knockdown of HDAC4 inhibits the proliferation of chicken SMSCs. (**A**) The expression level of cyclinD1, cyclinD2, Ki67, and PCNA was determined by qPCR in SMSCs after being transfected with si-HDAC4 and si-NC. (**B**) The proliferation activity of SMSCs after transfection with si-HDAC4 and si-NC was evaluated by CCK-8 at 12 h, 24 h, 36 h, and 48 h. (**C**,**D**) Flow cytometry for cell cycle analysis of SMSCs at 48 h after transfection of si-HDAC4 and si-NC. (**E**) EdU assays for SMSCs transfected with si-HDAC4 and si-NC for 36 h. EdU (red) fluorescence indicates proliferation. Hoechst (blue) fluorescence indicates nuclei. All photomicrographs are at 100× magnification. (**F**) The percentage of EdU stained cells to total cells was calculated at 36 h after transfection of si-HDAC4 and si-NC. In all panels, the values represent mean ± SEM from three independent experiments. * *p* < 0.05; ** *p* < 0.01.

To explore the role of HDAC4 on chicken SMSCs proliferation, SMSCs were transfected with si-HDAC4 and si-NC. Then, we detected the mRNA expression of four cell-proliferation-related genes Cyclin D1 (CCND1), Cyclin D2 (CCND2), a marker of proliferation Ki-67 (Ki67), and a proliferating cell nuclear antigen (PCNA). The results showed that the expression levels of CCND1, CCND2, and Ki67 were notably decreased in SMSCs transfected with si-HDAC4, compared to the si-NC transfected group (Figure 3A). In addition, as shown in Figure 3B, the results of CCK-8 showed that the cell vitality of the si-HDAC4 transfected group was obviously lower than that of the negative control group. Meanwhile, Cell cycle analysis showed that knockdown of HDAC4 decreased the cell population in the S and G2/M phase and significantly increased the cell population in the G1/G0 phase (Figure 3C,D). Additionally, EdU assay results showed that the numbers of EdU positive cells (proliferating cells) were significantly reduced after HDAC4 interference (Figure 3E,F). In summary, these results demonstrated that HDAC4 promoted the proliferation of SMSCs.

### 3.4. Knockdown of HDAC4 Inhibits the Differentiation of Chicken SMSCs

**Figure 4:**
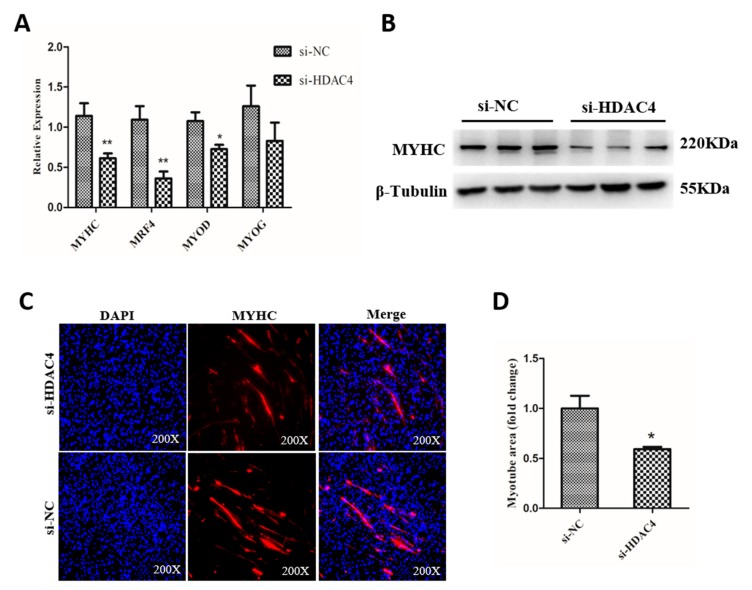
Knockdown of HDAC4 inhibits the differentiation of chicken SMSCs. (**A**) The mRNA level of MYHC, MRF4, MYOD1, and MYOG was determined by qPCR in SMSCs after being transfected with si-HDAC4 and si-NC. (**B**) The protein expression level of MYHC was determined by western blotting in SMSCs after being transfected with si-HDAC4 and si-NC. (**C**) SMSCs transfected with si-HDAC4 and si-NC induced to differentiate for 72 h were stained with MYHC antibody and DAPI (nuclei). All photomicrographs are at 200× magnification. (**D**) The percentage of myotube area to total cell area was calculated at 72 h after transfection of si-HDAC4 and si-NC. In all panels, the values represent mean ± SEM from three independent experiments. * *p* < 0.05; ** *p* < 0.01.

To determine the potential function of HDAC4 in SMSCs differentiation, we measured the expression level of muscle cell differentiation marker genes, including myosin heavy chains (MYHC), myogenic regulatory factor 4 (MRF4), myogenic differentiation 1 (MYOD1), and myogenin (MYOG) in SMSCs transfected with si-HDAC4 and si-NC. The results showed that the knockdown of HDAC4 significantly downregulated the mRNA levels of MYHC, MRF4, and MYOD1, compared to the si-NC transfected groups (Figure 4A). Additionally, the protein level of MYHC was detected by western blotting and the result was consistent with the qPCR results (Figure 4B). In addition, immunofluorescence assay results displayed that the relative myotube area of si-HDAC4 transfected group was notably less than that in the si-NC transfected group (Figure 4C,D). Together, our results suggest that HDAC4 promoted the differentiation of SMSCs.

### 3.5. Knockdown of HDAC4 Has No Effect on the Apoptosis of Chicken SMSCs

**Figure 5:**
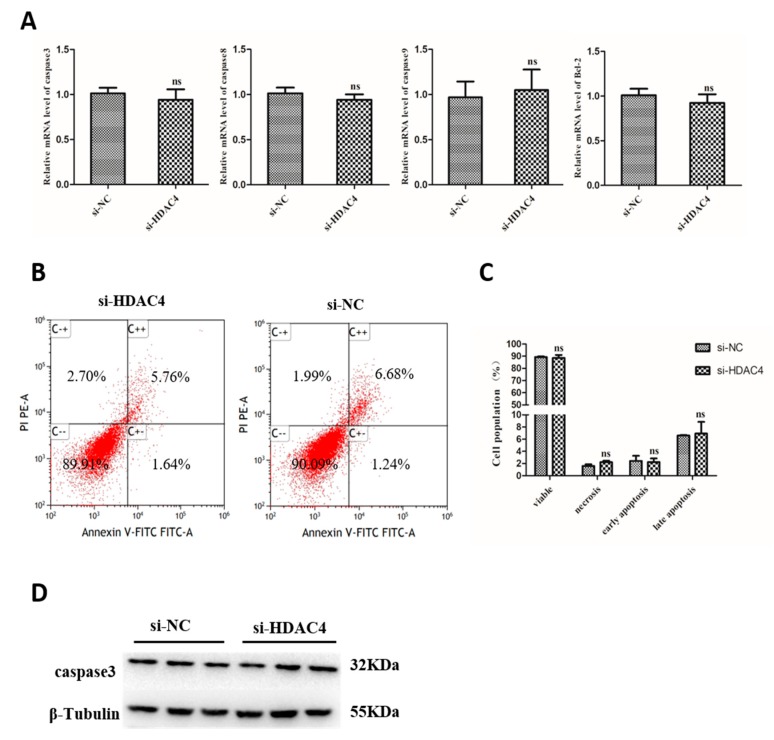
Knockdown of HDAC4 has no effect on the apoptosis of chicken SMSCs. (**A**) The mRNA level of caspase 3, caspase 8, caspase 9, and Bcl-2 was determined by qPCR in SMSCs after being transfected with si-HDAC4 and si-NC. (**B**,**C**) Flow cytometry for cell apoptosis analysis of SMSCs at 48 h after transfection with si-HDAC4 and si-NC. (**D**) The protein expression level of caspase 3 was determined by western blotting in SMSCs after being transfected with si-HDAC4 and si-NC. In all panels, the values represent mean ± SEM from three independent experiments. * *p* < 0.05; ** *p* < 0.01.

To evaluate the effects of HDAC4 on apoptosis of SMSCs, we detected the expression of Caspase-3, Caspase-8, Caspase-9, and Bcl-2 mRNA level in SMSCs that transfected the cells with si-HDAC4 and si-NC. However, we found that interference of HDAC4 had no significant effect on the expression of caspase-3, caspase-8, Caspase-9, and Bcl-2 (Figure 5A). In addition, flow cytometry was also used to detect cell apoptosis, the results showed that the apoptosis rates of si-HDAC4 and si-NC groups had no significant difference (Figure 5B,C). To further verify the effect of HDAC4 on SMSCs apoptosis, the protein level of caspase 3 was detected by western blotting, and the result was similar to that above (Figure 5D). In conclusion, our results suggest that HDAC4 had no significant effect on the apoptosis of SMSCs in chicken.

## 4. Discussion

Previous studies have shown that HDAC4 is involved in a variety of cellular functions, including chondrogenesis, osteoblast differentiation and chondrocyte hypertrophy, and muscle development [22,23]. The subcellular localization of HDAC4 in neurons is essential for neuronal activity [24,25]. In addition, HDAC4 also plays an important regulatory role in cancer, such as ovarian cancer [26], colon cancer [27], and B-cell lymphoma [28]. However, HDAC4 in myogenesis is less known, especially in poultry skeletal muscle development. Chickens are a well-established model to study skeletal muscle formation in vertebrates, as the developmental anatomy of chicken skeletal muscles share similarities to that of mammals, thus, we investigate the functions of HDAC4 in chicken SMSCs. In this study, we found that HDAC4 was highly expressed in skeletal muscles, meanwhile, HDAC4 was highly expressed at embryonic stages than that in postnatal muscles. Hence, we hypothesized that HDAC4 could regulate the growth and development of chicken skeletal muscle.

In order to explore the effect of HDAC4 on the proliferation, differentiation, and apoptosis in chicken SMSCs, we knocked down HDAC4 in chicken SMSCs. The results showed that the expression of proliferation-related genes (CCND1, CCND2, and Ki67) were significantly decreased, and cell vitality were inhibited and the proliferating cells ratio was significantly reduced. These results were consistent with the previous studies that Choi et al. found that the proliferation of HDAC4-deficient SMSCs assessed by BrdU were significantly reduced in mouse [29]. Additionally, the results of Nicoletta et al. showed that the number of HDAC4 knockout in SMSCs was prominently lowered, compared to the control group, and the expression of proliferation markers cyclin E1 and cyclin A2 were down-regulated in HDAC4-deficient SMSCs of mice [30]. Combined with the above results, we suggested that HDAC4 contributed to the proliferation of chicken SMSCs.

In the present study, knockdown of HDAC4 in differentiated SMSCs significantly inhibited the mRNA expression of MYHC, MRF4, and MYOD1, and decreased the protein level, compared to the control groups. Meanwhile, immunofluorescence showed that the formation of myotube was blocked. These results were similar to previous reports that HDAC4 could positively regulate the proliferation and differentiation of mouse SMSCs [29,30]. However, Wei et al. showed that the mRNA expression of MYHC, MYOD, and MEF2C was significantly increased after knocking down the HDAC4 in C2C12 cells [19]. As for the contradictory results, we think it was due to the different cell types but the results we got from three repetitions, were more congruent with that of Choi et al. and Marroncelli et al. Of course, different species can also be a source of difference in the results. These data showed that HDAC4 promoted the differentiation of chicken SMSCs.

Additionally, we found that the knockdown of HDAC4 had no significant effect on the apoptosis of SMSCs, based on the results of mRNA expression of caspase-3, caspase-8, caspase-9, and Bcl-2, the protein expression level of caspase-3, and the detection of the number of apoptotic cells by flow cytometry. This result was similar to that of Choi et al. who found that the relative mRNA level of caspase-3 was similar between the control and HDAC4 KO mouse SMSCs [29]. Thus, we suggested that HDAC4 has no significant effect on the apoptosis of chicken SMSCs.

## 5. Conclusions

In conclusion, we found that HDAC4 knockdown inhibits the proliferation and differentiation of chicken SMSCs, which suggested that HDAC4 might have an important role in the skeletal muscle growth and development of chickens.

## Figures and Tables

**Table 1:** Design of siRNA target on CDS of chicken HDAC4

siRNA	Sense Strands (5′→3′)	Anti-Sense Strands (5′→3′)
si-HDAC4-1107	GCAGGAUGCUGAGAGACUUTT	AAGUCUCUCAGCAUCCUGCTT
si-HDAC4-1790	GCAUUCACCAGCUGAGAAATT	UUUCUCAGCUGGUGAAUGCTT
si-HDAC4-2536	GCUUUCUACAAUGAUCCUATT	UAGGAUCAUUGUAGAAAGCTT
negative siRNA	UUCUCCGAACGUGUCACGUTT	ACGUGACACGUUCGGAGAATT

**Table 2:** Primers for qPCR.

Gene	Primer Sequences (5′→3′)	Product (bp)
HDAC4	F: CACAGCAAGTATTGGCATT	229
	R: CATCAGGTCGCTTCTCAG	
cyclin D1	F: CTCCTATCAATGCCTCACA	165
	R: TCTGCTTCGTCCTCTACA	
cyclin D2	F: GCACAACTTACTGACGATAG	125
	R: CTTCACAGACCTCCAACAT	
KI67	F: GCAACAACAAGGAGGCTTCG	93
	R: TTCAGGTGCCATCCCGTAAC	
PCNA	F: AACACTCAGAGCAGAAGAC	225
	R: GCACAGGAGATGACAACA	
MYHC	F: GAAGGAGACCTCAACGAGATGG	138
	R: ATTCAGGTGTCCCAAGTCATCC	
MRF4	F: GCGCCATCAGCTACATCGA	66
	R: GCATTTTGTCCTGCTGATCCA	
MYOD	F: GCCGCCGATGACTTCTATGA	66
	R: CAGGTCCTCGAAGAAGTGCAT	
MYOG	F: CGTGTGCCACAGCCAATG	63
	R: CCGCCGGAGAGAGACCTT	
caspase 3	F: TTCAGGTGCCATCCCGTAAC	186
	R: TCCACTGTCTGCTTCAATACC	
caspase 8	F: CCCTGAAGACAGTGCCATTT	106
	R: GGGTCGGCTGGTCATTTTAT	
caspase 9	F: GCTTGTCCATCCCAGTCCAA	95
	R: CAGTCTGTGGTCGCTCTTGT	
Bcl-2	F: ATCGTCGCCTTCTTCGAGTT	150
	R: ATCCCATCCTCCGTTGTTCT	
β-actin	F: GTCCACCGCAAATGCTTCTAA	78
	R: TGCGCATTTATGGGTTTTGTT	
