# Erratum: Zhao, J.; Shen, X.; Cao, X.; He, H.; Han, S.; Chen, Y.; Cui, C.; Wei, Y.; Wang, Y.; Li, D.; Zhu, Q.; Yin, H. HDAC4 Regulates the Proliferation, Differentiation and Apoptosis of Chicken Skeletal Muscle Satellite Cells. *Animals* 2020, *10*, 84

**DOI:** 10.3390/ani10122322

**Published:** 2020-12-07

**Authors:** Jing Zhao, Xiaoxu Shen, Xinao Cao, Haorong He, Shunshun Han, Yuqi Chen, Can Cui, Yuanhang Wei, Yan Wang, Diyan Li, Qing Zhu, Huadong Yin

**Affiliations:** Farm Animal Genetic Resources Exploration and Innovation Key Laboratory of Sichuan Province, Sichuan Agricultural University, Chengdu 611130, Sichuan, China; zhaojing@stu.sicau.edu.cn (J.Z.); shenxiaoxu@stu.sicau.edu.cn (X.S.); caoxinao@stu.sicau.edu.cn (X.C.); hehaorong@stu.sicau.edu.cn (H.H.); hanshunshun@stu.sicau.edu.cn (S.H.); chenyuqi@stu.sicau.edu.cn (Y.C.); cuican123@stu.sicau.edu.cn (C.C.); weiyuanhang@stu.sicau.edu.cn (Y.W.); as519723614@163.com (Y.W.); diyanli@sicau.edu.cn (D.L.); zhuqing@sicau.edu.cn (Q.Z.)

The authors wish to make the following corrections to their paper [1]:

In Figure 3, Figure 3C had identical images and has now been corrected (see the corrected version of Figure 3 below):

## Figures and Tables

**Figure 3:**
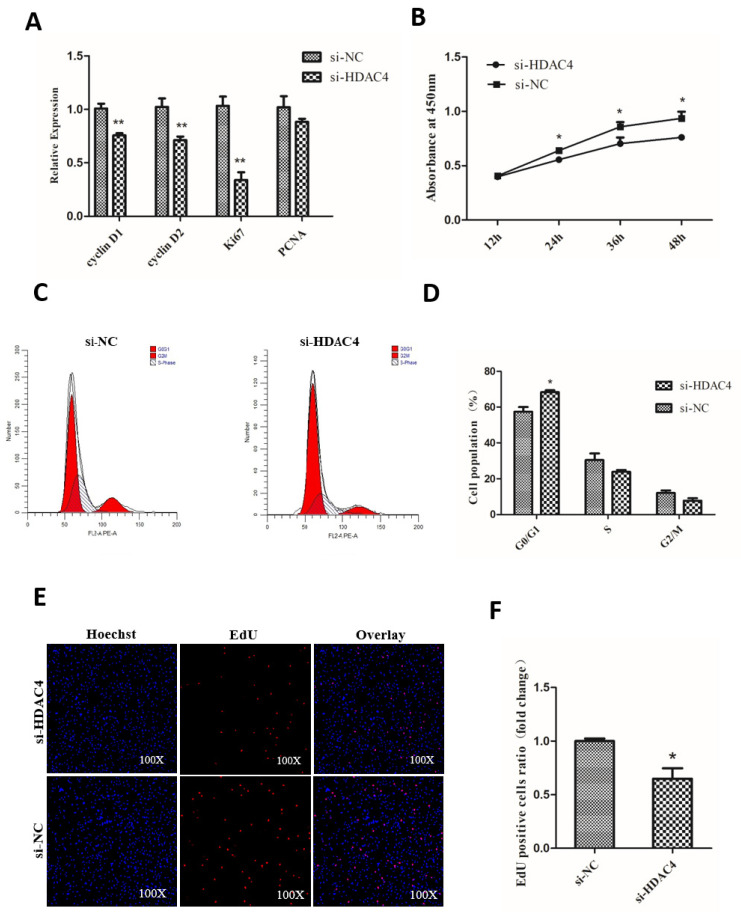
Knockdown of HDAC4 inhibits the proliferation of chicken SMSCs. (**A**) The expression level of cyclinD1, cyclinD2, Ki67, and PCNA was determined by qPCR in SMSCs after being transfected with si-HDAC4 and si-NC. (**B**) The proliferation activity of SMSCs after transfection with si-HDAC4 and si-NC was evaluated by CCK-8 at 12, 24, 36, and 48 h. (**C**,**D**) Flow cytometry for cell cycle analysis of SMSCs at 48 h after transfection of si-HDAC4 and si-NC. (**E**) EdU assays for SMSCs transfected with si-HDAC4 and si-NC for 36 h. EdU (red) fluorescence indicates proliferation. Hoechst (blue) fluorescence indicates nuclei. All photomicrographs are at 100× magnification. (**F**) The percentage of EdU stained cells to total cells was calculated at 36 h after transfection of si-HDAC4 and si-NC. In all panels, the values represent mean ± SEM from three independent experiments. * *p* < 0.05; ** *p* < 0.01.
